# Multi-Regional Study on the Microbial Community Structure, Core Microbiome and Functional Characteristics in Deep Fracture Waters

**DOI:** 10.3390/microorganisms14010045

**Published:** 2025-12-25

**Authors:** Xiaoxuan Li, Tianming Huang, Yiman Li, Zhonghe Pang, Yuran Zhang

**Affiliations:** 1 State Key Laboratory of Lithospheric and Environmental Coevolution, Institute of Geology and Geophysics, Chinese Academy of Sciences, Beijing 100029, China; lixiaoxuan@mail.iggcas.ac.cn (X.L.); tmhuang@mail.iggcas.ac.cn (T.H.); liyiman@mail.iggcas.ac.cn (Y.L.); z.pang@mail.iggcas.ac.cn (Z.P.); 2 College of Earth and Planetary Sciences, University of Chinese Academy of Sciences, Beijing 101408, China

**Keywords:** deep fracture water, microbial community, core microbiome, environmental filtration, meta-analysis

## Abstract

The deep terrestrial subsurface is the largest reservoir of Earth’s freshwater resources as well as the largest habitat for prokaryotic life. However, the deep-subsurface microbiome, especially its spatial distribution across countries/continents, is still poorly understood. In this study, we compiled and compared 30 16S rRNA gene amplicon libraries from three deep fractured aquifers in different parts of the world (depth range of tens of meters to 2.4 km below surface) to understand the spatial distribution and functions of deep-subsurface microbial community, and to test for the presence of core taxa. The results revealed spatially heterogenous microbial community composition at both the local and the global scales, even at the phylum level. Environmental filtering was identified as an important driver of the microbial community structure of deep groundwaters. Despite the spatial heterogeneity, the three aquifers share a core microbiome at the genus level. Only one family, *Comamonadaceae*, was present in all the 30 samples analyzed. Several other families were also prevalent, including *Hydrogenophilaceae*, *Omnitrophaceae*, *BSV26 (Candidatus Kryptonia)*, and an unclassified *Thermodesulfovibrionia*. FAPROTAX functional prediction indicated that chemoheterotrophic functions predominate, and the core microbial genera, together with the dominant genera, collectively govern the functional characteristics. Taken together, our findings provide new insights into the spatial heterogeneity and functional potential of deep-subsurface ecosystems across the globe.

## 1. Introduction

The deep terrestrial subsurface (hundreds to thousands of meters below the Earth’s surface) is now recognized as the largest habitat of prokaryotic life on Earth [1,2]. Its unique physicochemical conditions, such as high temperature, high pressure, nutrient depletion, and dark anaerobiosis, foster diverse microbial communities with specialized functions [3,4,5]. Groundwater in deep, fractured aquifers is not only a core habitat for microbial survival but also a vital carrier for material cycling and energy transfer. The composition and function of groundwater microbial community directly influence the biogeochemical cycles of carbon, nitrogen, sulfur, and other elements in the deep subsurface.

Despite recent advancements in high-throughput sequencing technology that have promoted research in deep subsurface microbiology, studies on microbial communities in deep fractured aquifers still face numerous challenges compared to those in surface and shallow environments. On the one hand, the difficulty and high cost of obtaining deep subsurface samples result in limited high-quality datasets in the literature. On the other hand, the geological backgrounds (e.g., lithology, structure) and hydrogeochemical conditions (e.g., pH, redox potential, dissolved nutrient content) of deep strata vary substantially among regions. This has hindered the formation of a unified understanding regarding the spatial distribution patterns, driving mechanisms, and the core functional taxa of deep-subsurface microbial communities [6]. In particular, the existence and ecological functions of the “core microbiome” is a hot topic and point of contention in current microbial ecology research [7]. The “core microbiome” is considered to be the key taxon adapted to the common selective pressures of specific habitats, and its presence or absence directly reflects the degree of habitat homogenization and microbial adaptive strategies [8,9]. Furthermore, the coupling relationship between the functional potential of deep-subsurface microorganisms (e.g., organic matter degradation, distribution of functional genes related to element cycling) and their associated hydrogeochemical conditions requires further verification through systematic data analysis and functional prediction [10].

To date, most deep-subsurface microbiome research had focused on individual regions [11,12,13,14], or addressed it solely from the perspective of geochemistry [15] or microbial diversity [16], lacking an overarching understanding of the whole picture. Furthermore, because different research groups often employ different strategies in sequencing data processing and data visualization, the results across different 16S-based microbiome studies are almost impossible to compare, necessitating meta-analyses based on their raw sequencing data [17,18]. To address the aforementioned research gaps, in this study we analyzed the 16S rRNA gene amplicon sequencing data in conjunction with hydrogeochemical data from three deep, fractured aquifers across the globe. The main objectives of this study are (1) to find out if the hydrogeochemical properties of deep fracture waters are heterogenous across different geographical regions; (2) to analyze the diversity, abundance distribution, and similarity patterns of microbial communities in deep fracture waters; (3) to find out if a core microbiome exists in deep aquifers across different continents; and (4) to predict the functional potential of deep aquifer microbial communities and evaluate the effect of hydrogeochemical conditions on their structures and functions.

## 2. Materials and Methods

### 2.1. Study Area

**Figure 1:**
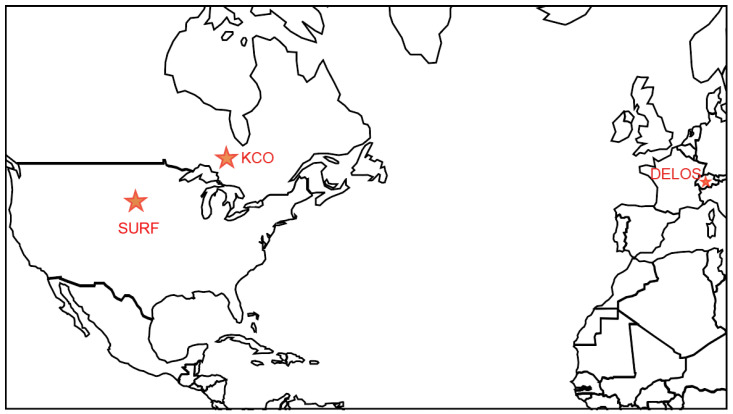
Geographical locations of the underground laboratories where the samples analyzed in this study were obtained from.

Three deep-subsurface aquifers (Figure 1) were selected in this study, accessed through the BedrettoLab Deep Life Observatory (DELOS), the Kidd Creek Observatory (KCO) and the Sanford Underground Research Facility (SURF), respectively. The DELOS is located in the Riale di Ronco high-altitude Alpine catchment of the Gotthard Massif, Switzerland, accessible via the Ronco Portal of the 5.2 km long Bedretto Tunnel (46.497518° N, 8.494992° E) that is tens of meters to 1.6 km underground [19]. Its lithology includes three geological units [20,21]: the Tremola Series (tunnel meter (TM) 0–434), composed of mica-gneiss, amphibolites, schists, calc-silicate rocks, and quartzites [22]; the Prato Series (TM 434–1138), dominated by mica-gneisses, amphibolites, and schists with centimeter-to-meter scale compositional heterogeneity; and the Rotondo granite (TM 1138–5218), further divided into equigranular granite (RG1) and biotite-rich porphyritic granite (RG2) with zircon U-Pb intrusion ages ranging from 280 to 335 Ma [20]. The KCO is situated 2.39 km (7850 ft) underground in an active copper-silver-zinc mine of the 2.7 Ga Canadian Shield near Timmins (Ontario, Canada) within the traditional territories of the Anishinabewaki and Cree Nations. Its lithology is dominated by rhyolitic layers bounded by high-silica rhyolite flows, tholeiitic basalts, and up to 500 m-thick carbonate-altered komatiitic flows that host the 2.7 Ga Kidd Creek massive sulfide deposit [23]. The SURF, formerly the Homestake Gold Mine, is situated in Lead, SD, USA, within the northern Black Hills region. The samples analyzed here were obtained from the 4850 ft (1478 m) underground level of SURF, within the Precambrian Poorman Formation dominated by sericite-carbonate-quartz phyllite [24].

### 2.2. Data Acquisition

To evaluate the community composition and functional potential of the deep subsurface fracture water microbiome, four published microbial assessment studies were selected based on the following criteria: (1) Fluid samples originated from deep-subsurface fractured aquifers. (2) Publicly available 16S rRNA gene amplicon sequence data. (3) Amplification of the V4 and V5 hypervariable regions of the 16S rRNA gene was performed using the universal primer pair 515F-Y (5′-GTGYCAGCMGCCGCGGTAA)/926R (5′-CCGYCAATTYMTTTRAGTTT) [25]. (4) High-throughput sequencing was performed using the Illumina Miseq platform (Illumina, San Diego, CA, USA). Only a subset of representative samples from each study were selected (15 samples from SURF, 14 samples from DELOS and 1 sample from KCO), covering as much microbial variety as possible using only a small number of samples (30 samples total).

Raw high-throughput 16S rRNA gene amplicon sequence data sets for the SURF groundwater were obtained from the European Nucleotide Archive at the European Bioinformatics Institute, under accession no. PRJEB35125 [26] and PRJEB44691 [27]. The former accession no. corresponds to pristine groundwater samples obtained from artesian boreholes and dripping fractures including BoreholePST, BoreholeGC, Port17Ledge, FractureA, FractureB and FractureC [26], whereas the latter accession no. corresponds to time-series produced water samples from a 10-month injection test at a geothermal test site, named according to the date each sample was taken: PB/PDT/PI/PST-MMDDYY [27,28]. Raw amplicon sequence data for the DELOS were obtained from the National Center for Biotechnology Information’s (NCBI) under accession no. PRJNA1181539 [19]. This accession no. corresponds to pristine groundwater samples obtained from flowing fractures (TM1306, TM1494, TM2647, TM2848, TM4166, TM4348, TM4447, TM444, TM4652, TM4752, TM4846 and TM5132) and uncased boreholes (TM901, TM2794), named according to the horizontal distance from the tunnel entrance at which each sample was taken: TM-XXXX. Raw amplicon sequence data for the KCO were obtained from the European Nucleotide Archive at the European Bioinformatics Institute, under accession no. PRJEB49237 [29]. This accession no. corresponds to pristine groundwater samples obtained from artesian boreholes including KC12299.

Note that the samples collected on 16 May 2019 and 11 December 2019 in the SURF time-series produced fluid dataset were selected in this study because the community compositions differed significantly between these two dates in each well. We aimed to capture as much community variation as possible within each study area using the minimum number of samples. One exception is PB_082819, whose community structure was similar with that of PB_121119. PB_082819 was included just as a reference. For the DELOS dataset, we selected one sample per tunnel meter. For each location with time-series data available (TM444, TM1306, TM1494, TM2848 and TM4652), only the last sample of the time series was selected. For the KCO dataset, only samples from one borehole named 12299 was deemed representative of the aquifer, which is why only one sample “KC12299” was selected from KCO.

Hydrogeochemical data for the three regions were obtained from the US Department of Energy Geothermal Data Repository (at https://gdr.openei.org/submissions/1424, accessed 1 October 2025), https://github.com/GeobiologyLab/DELOS-2021-time-series (accessed 1 October 2025), and the Interdisciplinary Geoscience Data Alliance (at https://doi.org/10.60520/IEDA/113522, accessed 1 October 2025), respectively.

### 2.3. High-Throughput Sequencing Data Processing

SURF: Primer sequences were trimmed from the raw sequencing reads of each sample using cutadapt (version 5.2) [30]. The Dada2 (version 1.34.0) [31] and phyloseq (version 1.50.0) [32] packages in R were used to analyze the sequencing data. Sequence quality filtering was performed by truncating all reads exceeding 220 base pairs (discarding bases with quality scores < 30) and removing sequences that did not perfectly match the proximal primers, contained more than two expected errors, or harbored ambiguous bases (Ns). The Dada2 [31] method was used to infer Amplicon Sequence Variants (ASVs) from quality-filtered reads, remove sequencing errors, merge forward and reverse reads while disallowing mismatches in overlapping regions, remove chimeras, and generate a ASV table.

DELOS: All data processing procedures were conducted on the Galaxy online bioinformatics cloud platform (https://usegalaxy.org). Based on the sequence quality, the first 5 nucleotides of the forward and reverse reads were trimmed using the FASTQ Trimmer by column (Galaxy Version 1.2+galaxy0) [33,34]. Meanwhile, the last 21 nucleotides of the reverse read were trimmed using FASTQ Trimmer by column. After trimming, Trimmomatic (Galaxy Version 0.39+galaxy2) [35] was used for quality filtering of the reads (SLIDINGWINDOW:100:28), and adapters (TruSeq3, paired—ended, for MiSeq and HiSeq) were removed. Paired end reads were joined using fastq-join [36] (-p 3 -m 20). The resulting data were downloaded, and an ASV table was generated in R using the Dada2 package.

KCO: Sequence quality filtering was carried out the same as for the SURF dataset, with the sole modification that reads exceeding 230 bp were truncated.

The above data processing method was selected to maintain consistency with the data processing approach in the original publications. Finally, the sequence tables generated for each dataset were merged, with duplicate ASVs resolved to produce a ASV count matrix for subsequent analysis. ASVs were annotated using the dada2 package and the Silva nr99 v138.2 dataset [37,38,39].

### 2.4. Water Chemistry Data Processing

Bicarbonate data was not available in some of the original publications. Therefore, based on a measured pH of 7~9.5 for all samples, bicarbonate concentrations were calculated via charge balance, with the assumption that the net positive charge of the samples is fully offset by bicarbonate [40].

### 2.5. Diversity and Statistical Analyses

The diversity and statistical analyses were performed using R packages phyloseq (1.50.0) [32], vegan (2.7.2) [41], and NbClust (3.0.1) [42]. Shannon and Observed indices were used to study alpha diversity. For beta diversity analyses, we calculated community similarity at both the phylum and the ASV level using both the Bray–Curtis and weighted Unifrac metrics. For the Bray–Curtis metric, the computation was implemented using the distance() function in the R package phyloseq, with the parameter method = “bray” specified to generate the Bray–Curtis distance matrix. The optimal number of clusters was determined by silhouette analysis [43] on the Bray–Curtis dissimilarity matrix [44] using the NbClust() (min.nc = 2, max.nc = 10, method = “ward.D”, index = “silhouette”) from NbClust (version 3.0.1) package [42]. For the weighted UniFrac metric, ASV-level analyses were performed using *method* = “*wunifrac*”, with all other parameters identical to those used for the Bray–Curtis metric. At the phylum level, ASVs were first agglomerated to the phylum rank using tax_glom, after which the same procedures as in the ASV-level analysis were applied. The VennDiagram (version 1.7.3) [45] package in R was used to draw Venn diagrams.

### 2.6. Functional Prediction Analyses

The ASV table was first agglomerated at the genus level to obtain the number of reads each genus has in each sample. Then, the abundance of each function in each sample was calculated by summing the reads of those genera that contains the function in the database. Note that the ASVs not classifiable at the genus level was not considered in the FAPROTAX analysis. The above data analysis was performed using the Majorbio Cloud (www.majorbio.com) [46,47]. Functional aggregation was performed using the official collapse_table.py script [48] provided by FAPROTAX (v1.2.1) [48,49], generating an abundance table of different functional categories across all samples. Subsequently, the functional abundance table was exported, and the abundance of each function was divided by the total number of functional reads in each sample to obtain the relative abundance of each function in each sample. Finally, the packages “Complex Heatmap (version 2.22.0)” and “vegan (version 2.7.2) ” in R were used to generate the heatmap of functional relative abundance. For each functional category, the relative abundance values were standardized to z-scores [50] with a mean of 0 and a standard deviation of 1. Clustering was performed using the Bray–Curtis dissimilarity at the genus level for samples (columns) and Euclidean distance for functional genes (rows).

## 3. Results

### 3.1. The Hydrochemical Properties of Deep Fracture Waters Are Spatially Heterogenous Across Study Regions

**Figure 2:**
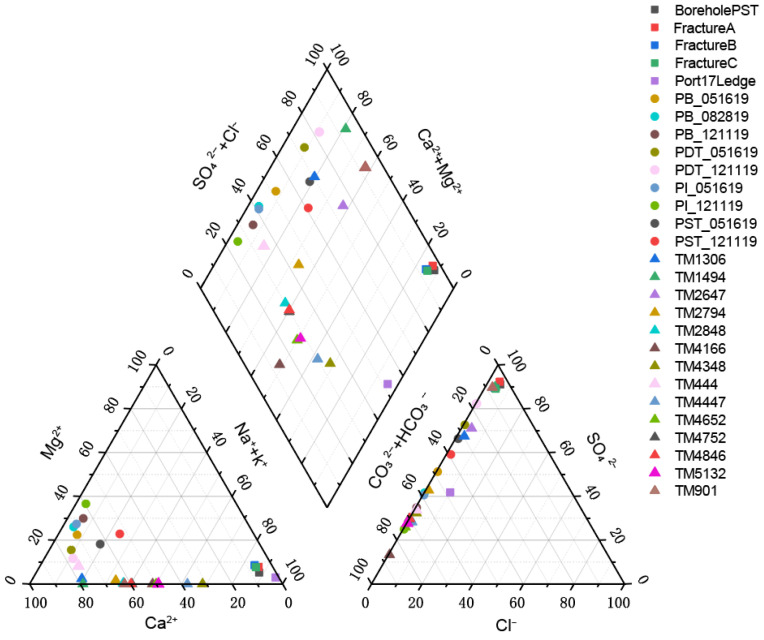
Hydrochemical composition of the groundwater samples analyzed in this study displayed in a Piper diagram.

The pH of the three selected aquifers ranges from 7 to 9.5, indicating a weakly alkaline environment. A Piper diagram was generated based on the water chemistry data from each aquifer (Figure 2, Table S1). The hydrochemical types in the SURF region exhibit distinct patterns depending on whether the sampled water was pristine or not. The pristine groundwater samples from artesian boreholes and dripping fractures, including Borehole PST, FractureA, FractureB and FractureC almost completely overlapped on the lower-right corner of the cation diagram and on the upper corner of the anion diagram, indicating that they had very similar geochemical compositions, corresponding to the SO_4_-Na·K hydrochemical type. In contrast, the time-series produced water samples exhibit a scattered distribution in the lower-left region of the cation diagram. On the anion diagram, the time-series data points are distributed along the HCO_3_^−^ axis, dominated by HCO_3_^−^ and SO_4_^2−^ ions. Based on these geochemical signatures, the time-series data points are therefore classified as either the HCO_3_-Ca·Mg or HCO_3_·SO_4_-Ca·Mg hydrochemical type. The hydrochemical composition of deep fissure water in the DELOS region exhibits higher dispersion. On the cation diagram, data points are distributed along the Ca^2+^ axis, dominated by Ca^2+^ and Na^+^/K^+^. However, the anion diagram exhibits distinct banding patterns: the TM901 and TM1494 samples overlap almost entirely in the upper corner, indicating sulphate-rich water types; the TM1306 and TM2647 samples show remarkable similarity, with SO_4_^2−^ predominating alongside increased HCO_3_^−^; The proportion of HCO_3_^−^ in TM2794–TM5132 generally increases with depth into the tunnel, consistent with the original findings [19]. ln particular, the TM4166 sample occupies the lower left corner, where HCO_3_^−^ is strongly dominant. The KCO region was not plotted on the Piper diagram due to the presence of only one sample, KC12299, with incomplete water chemistry data. However, previous studies [29,51,52,53] have revealed that the deep fracture water in this region is typically characterized by high salinity, as exemplified by sample KC12299 with a total dissolved solids (TDS) content of 197,274 mg·L^−1^ (Measured value), corresponding to the Ca-Cl hydrochemical type. TDS ranged from 40–670 mg·L^−1^ (Measured value) in DELOS. Since SURF did not include measured TDS values, TDS was estimated using an empirical calculation formula and was calculated by

$$TDS=\sum \left(anions\right)+\sum \left(cations\right)-\frac{1}{2}\left[{HCO}_{3}^{-}\right]$$

where the symbol in the parentheses denoted the mass concentration of the dissolved ion, with the unit of mg·L^−1^. The results showed that the TDS content in the SURF area ranged from 530 to 6700 mg·L^−1^. This calculation approach has inherent limitations, including incomplete coverage of all components contributing to TDS and the potential amplification of uncertainties associated with ion measurements. Therefore, the results should be regarded as indicative rather than definitive.

### 3.2. Different Microbial Community Compositions Within-Site and Across-Sites

**Figure 3:**
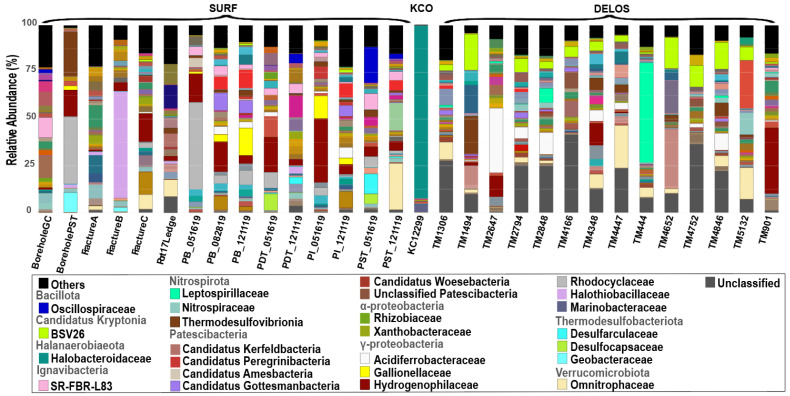
The microbial community composition of the 30 groundwater samples analyzed in this study. Bar plots show the finest classification possible down to the family level. The major taxa (i.e., taxa that were within the top 10 most abundant in at least one sample) are shown in color. The complete legend is shown in Figure S1.

A total of 2486 ASVs were inferred from the selected SURF sequencing dataset. These ASVs encompassed 68 phyla and 254 families in total. A total of 25 ASVs were inferred from the selected KCO sequencing dataset. These ASVs encompassed 7 phyla and 19 families in total. A total of 6063 ASVs were inferred from the selected DELOS dataset. The number of raw reads, the library size after quality filtering, and the number of ASVs for each sample are summarized in Table S2. These ASVs encompassed 69 phyla and 248 families in total. The microbial community compositions of the total of 30 selected samples are shown in Figure 3. It can be seen that a number of taxa were present in multiple samples across different aquifers.

**Figure 4:**
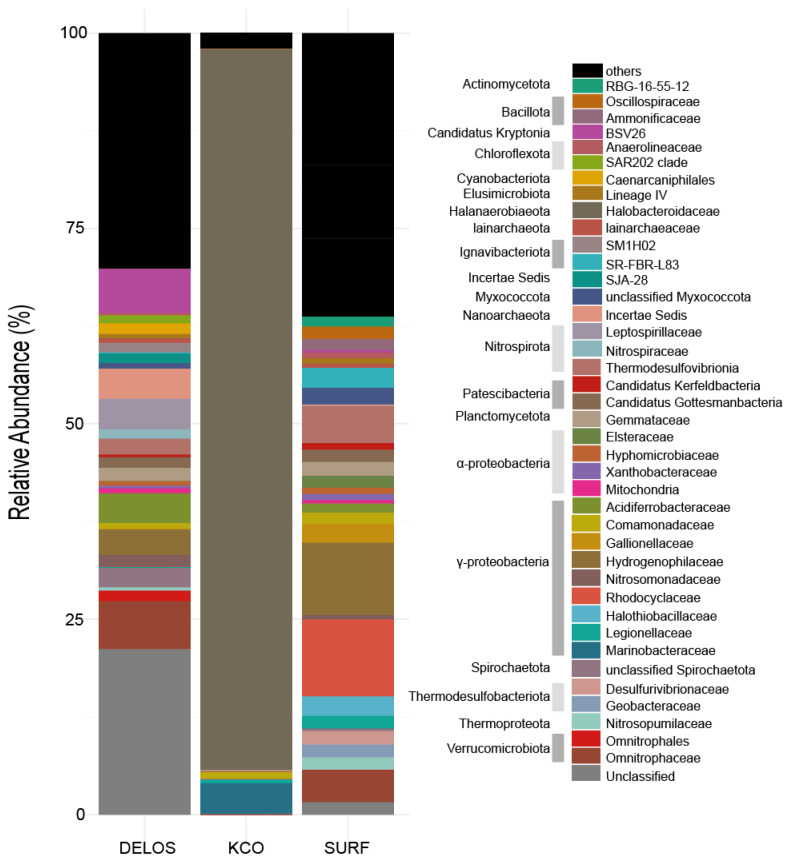
Averaged microbial community profile in the sample set across three regions. Only the major taxa (top 40 most abundant y across wells in the averaged microbial community profile) are shown in color. For taxa not classifiable at the family level, the finest possible classification is shown.

In terms of community dominance and structural complexity, the three regions exhibited significant differences in their average community characteristics (Figure 4). The average relative abundance of the one sample from the KCO region indicates it almost entirely consists of one bacterial phylum and its corresponding family: *Halanaerobiaeota* (phylum) and *Halonotobacteriaceae* (family), the latter accounting for 92.2% of the total abundance. All other taxa including *α-Proteobacteria*, *γ-Proteobacteria*, *Actinobacteria* and *Planctomycetes* are present at abundances below 6%, resulting in a “single-taxon-dominated” [54] community feature. In contrast, DELOS and SURF display more complex and diverse community structures. As shown in Figure 4, DELOS is characterized by a balanced distribution of multiple dominant taxa: the three most abundant phyla (*Pseudomonadota*, *Nitrospirota*, and *Verrucomicrobiota*) collectively represent 37.4% of the total abundance, and the three most abundant families (*BSV26*, *Omnitrophaceae* and *Acidiferrobacteraceae*) sum to 17.8%. No single taxon in DELOS exceeds 15% abundance, indicating a “multi-taxon-co-dominant” pattern [55]. SURF, meanwhile, exhibits a “few-dominant-taxa-led” structure: its three most abundant phyla (*Pseudomonadota*, *Thermodesulfobacteriota* and *Bacillota*) account for 59.3% of the total abundance, and the three most abundant families (*Hydrogenophilaceae*, *Rhodocyclaceae* and *Halothiobacillaceae*) contribute 22%.

### 3.3. Alpha Diversity Analysis

**Figure 5:**
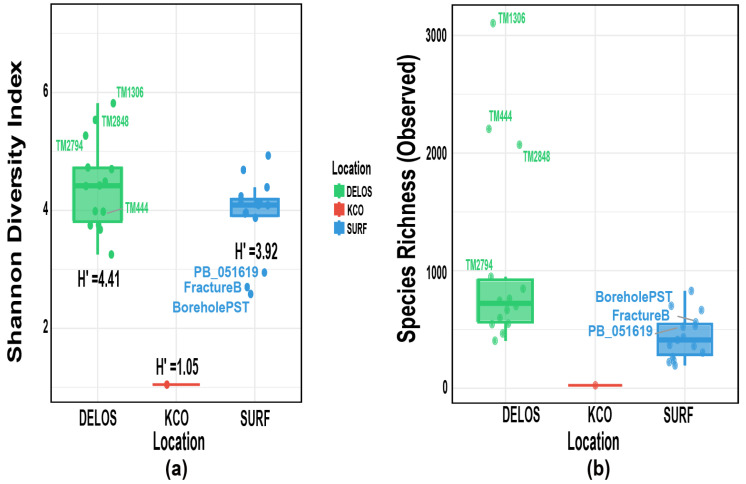
Box plots of alpha-diversity metrics: (**a**) Shannon diversity index; (**b**) Observed species richness. The vertical lines indicate the boundaries of the data distribution, and the horizontal lines from bottom to top represent the minimum boundary (Q1 − 1.5 × IQR), the first quartile (Q1, 25th percentile), the median (50th percentile), the third quartile (Q3, 75th percentile), and the maximum boundary (Q3 + 1.5 × IQR). Note that IQR = interquartile range. H’ represents the mean value of the Shannon–Wiener diversity index.

Alpha diversity generally refers to metrics that describe species richness, evenness, or the inherent diversity of a given sample [56]. The Shannon alpha diversity index (H’) was calculated for the three study regions (Figure 5a). Among them, the DELOS region exhibited the highest mean diversity (H’ = 4.41), followed by the SURF region (H’ = 3.92), while the KCO region showed the lowest value (H’ = 1.05). These results indicate that the microbial community structure in the DELOS region is relatively more diverse and uniform, whereas the KCO region has the lowest community diversity, which is consistent with the relative abundance profile of each region. Consistent with the elevated Shannon indices (Figure 5a), observed species richness (Figure 5b) was also exceptionally high in DELOS samples TM1306 and TM2848. Interestingly, TM444, BoreholePST, and FractureB exhibited high observed richness but low Shannon values, indicating dominance by a few taxa, a pattern consistent with the corresponding relative-abundance profiles.

### 3.4. Samples from Different Regions Form Distinct Clusters

**Figure 6:**
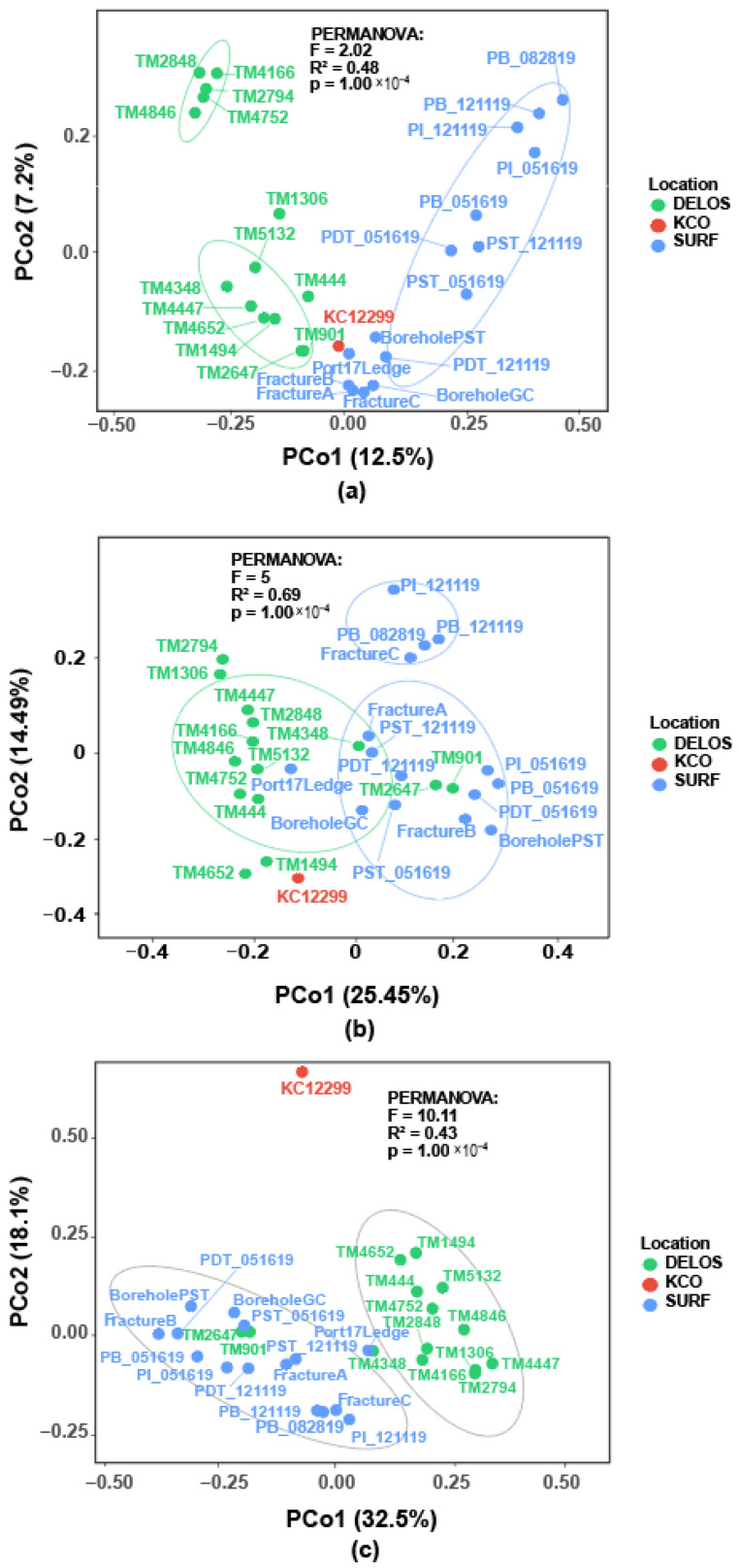
PCoA analysis plots of microbial community datasets across the three regions: (**a**) Based on the Bray–Curtis distance at the ASV level; (**b**) Based on the weighted Unifrac distance at the ASV level; (**c**) Based on the weighted Unifrac distance at the Phylum level.

To assess the beta diversity of microbial communities in the deep subsurface, Principal Coordinate Analysis (PCoA) was performed using both Bray_Curtis and weighted Unifrac distance metrics. (Figure 6). The Bray–Curtis distance was calculated between each pair of samples at the ASV level (Figure 6a). The first two principal coordinates (PCo1 and PCo2) explained 12.5% and 7.2% of the total variance, respectively, with samples from different sampling sites (DELOS, KCO, and SURF) forming distinct clusters. The DELOS samples (green dots) mostly clustered in the left-hand region of the plot, forming two relatively compact subclusters. This indicates a certain degree of dissimilarity in microbial community composition within each subcluster at this sampling site. SURF samples (blue dots) were distributed across the right-hand region, forming a cluster distinct from that of the DELOS samples. This suggests significant differences in microbial community composition between SURF and DELOS sites. On the other hand, the distribution of data points of the SURF produced-fluid samples was relatively dispersed, with greater inter-individual distances between time-series produced waters than between pristine groundwater samples. The KCO sample (red dot) occupy a position between the DELOS and SURF clusters, suggesting their microbial community composition possesses unique characteristics while exhibiting associations with both other sites. Naturally, the limited sample size of KCO must be considered as a potential confounding factor, requiring further validation of its community features with additional data. Furthermore, PERMANOVA analysis (F = 2.02, R^2^ = 0.48, *p* = 1.00 × 10^−4^) further confirmed statistically significant differences in microbial β-diversity across distinct deep subsurface locations. This result could be an indicator of the significant influence of unique subsurface environmental conditions on shaping the microbial community structure.

Principal coordinate analysis (PCoA) based on the weighted Unifrac distance reveals notable segregation between the DELOS (green) and the SURF (blue) communities along the first principal coordinate (PCo1) (Figure 6b). This pattern indicates significant differences in groundwater community structure between the two regions. However, partial overlap between clusters suggests the presence of shared core microbial taxa. Collectively, PCo1 and PCo2 explained approximately 40% of the total variance, representing the primary axes of microbial community differentiation within the study areas. Upon taxonomic agglomeration to the phylum level, samples from the three regions again formed visibly distinct clusters (Figure 6c), highlighting across-region community heterogeneity even at the phylum level (Figure S2). PERMANOVA indicated that sampling location exerted a significant effect on microbial community structure (F = 10.11, R^2^ = 0.43, *p* = 0.0001), underscoring geography as a key factor shaping phylogenetically weighted community composition.

### 3.5. Limited Shared Microbial Taxa Among Three Regions

**Figure 7:**
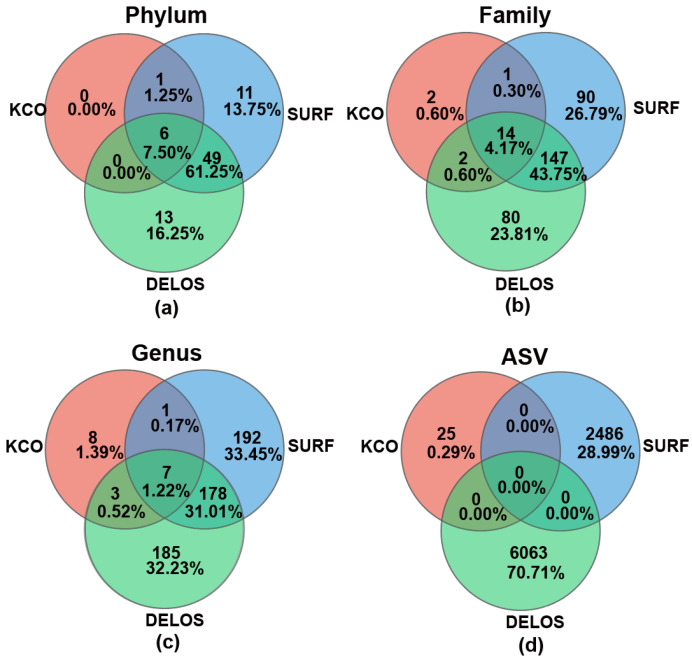
Venn diagram analysis on the microbial taxa across different regions at the (**a**) phylum level, (**b**) family level, (**c**) genus level, and (**d**) ASV level.

Venn diagram analysis was used to interrogate the shared microbial taxa among different regions. Six phyla (*Pseudomonadota*, *Planctomycetota*, *Bacillota*, *Chloroflexota*, *Actinomycetota*, and *Deinococcota*) were common to all three regions (Figure 7a, Figure S2 and Table S3), representing 7.50% of all phyla detected in this study. 49 phyla (61.25%) were shared exclusively between SURF and DELOS, among which *Nitrospirota*, *Patescibacteria*, *Verrucomicrobiota*, and *Candidatus Kryptonia* exhibited markedly higher relative abundances. The three most abundant phyla exclusive to the SURF region were *Deferribacterota*, *Campylobacterota*, and *FW113*, whereas the three most abundant phyla exclusive to the DELOS region were *Candidatus Lindowbacteria*, *Firestonebacteria*, and *Abditibacteriota*. In contrast, KCO contained only 7 phyla (8.75%), although the small number of phyla could be the result of the insufficient sample size. At the family level (Figure 7b), 14 families were common to all three regions, representing 4.17% of all the families detected across the three aquifers. Notably, SURF and DELOS harbored a shared core microbiome of 147 families, which constituted 43.75% of the combined family pool (i.e., the total number of unique families) across the three regions. A total of 7 shared genera were detected across the three regions, accounting for 1.22% (Figure 7c). At the ASV level, no shared nucleotide sequences were identified (Figure 7d), although this could be an artifact of the different bioinformatics pipelines used and PCR amplification bias across different aquifers.

### 3.6. FAPROTAX Functional Prediction Reveals Metabolic Niche Partitioning of Groundwater Microbiota in C–N–S–Fe Cycling Across the Three Study Regions

FAPROTAX is a functional annotation database that establishes associations between bacterial/archaeal taxa and metabolically/ecologically relevant functions (e.g., nitrogen fixation, sulfate respiration, and hydrocarbon degradation) based on the literature documenting cultured representatives [57]. This database was originally constructed for a study focusing on marine environments, incorporating 80+ functional groups and taxonomic details corresponding to over 4600 bacteria and archaea from oceanic habitats [48,58]. Numerous existing studies have employed FAPROTAX for the functional annotation of groundwater prokaryotes, which has verified the reliability of its annotation results and supported its applicability in this field [59,60,61,62].

**Figure 8:**
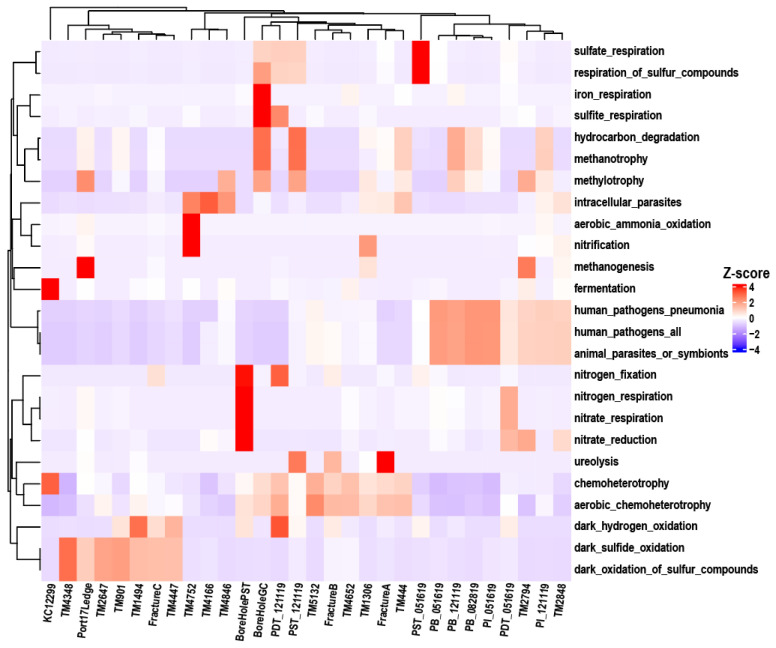
FAPROTAX functional profiles. (only the top five most abundant functions per individual sample are considered.) Clustering among functions are performed based on Euclidean distance, whereas clustering among samples are conducted using Bray–Curtis distance.

In this study, a grouping analysis was conducted on 12,264 target records, with 1871 out of 12,264 records (15.26%) assigned to at least one group, and 61 groups were represented (i.e., associated with at least one record). FAPROTAX functional prediction based on 16S rRNA gene sequences indicated that the groundwater microbial communities in the three regions all possessed the metabolic potential to drive the biogeochemical cycling of key elements such as C, N, S, and Fe (Figure 8). Overall, chemoheterotrophy was the shared and most abundant functional category in the system, and aerobic chemoheterotrophy accounted for a significant proportion in some samples.

Regarding inter-regional differences, the relative abundances of sulfate_respiration, respiration_of_sulfur_compounds in SURF samples were significantly higher than those in DELOS and KCO, indicating an anaerobic habitat rich in sulfate where more active sulfur cycling processes may have occurred. In oxygen limiting environments, many aerobic bacteria are capable of switching from aerobic to anaerobic respiration to generate energy. Nitrate respiration is a metabolic process in which microorganisms use nitrate as the terminal electron acceptor under anaerobic conditions [63]. The relatively high abundances of nitrogen fixation, nitrate reduction, nitrate respiration, and nitrogen respiration in the BoreHolePST sample reflect the efficient adaptation of microorganisms to the environment characterized by limited oxygen availability and relatively scarce energy substrates. Meanwhile, BoreholeGC exhibited relatively high abundance of iron_respiration, which is consistent with the elevated iron concentration reported in a previous study [26]. The TM901, TM2647, and TM4348 taxa in DELOS showed relative advantages in dark_oxidation_of_sulfur_compounds and dark_sulfide_oxidation, suggesting a dark/low-light microhabitat with sufficient sulfide and available electron acceptors. In addition, fermentation was only highly abundant in KCO, indicating an hypoxia/severe hypoxia habitat with abundant fermentable organic matter and limited electron acceptors. Functional labels related to human or animal pathogenesis, specifically human_pathogens_all and animal_parasites_or_symbionts, showed relatively high abundances in some samples. This suggests that the associated pathogens are present not only in their hosts, but in groundwater systems as well, necessitating further quantitative PCR analysis to evaluate their ecological risks.

Functional-level clustering revealed that most samples exhibited highly similar patterns between taxonomic abundance and functional abundance, indicating a generally positive correlation between microbial community composition and its potential ecological functions. However, a few samples (e.g., FractureC, TM4447, and TM1494) were placed on distant branches in the taxonomic dendrogram but clustered closely in the functional tree, suggesting possible niche convergence.

## 4. Discussion

### 4.1. Environmental Filtration Exerts a Driving Influence on the Microbial Community Structure Within Deep Groundwater Environments

The KCO, SURF, and DELOS sites are distinguished by contrasting hydrochemical conditions. In the KCO area, the groundwater exhibits exceptionally high mineralization, which may be attributed to its prolonged residence time. During long-term water–rock interactions [29] and geochemical evolution, the groundwater gradually evolved into a Ca–Cl hydrochemical type. The SURF area was historically a gold mining district and retains abundant sulfide minerals (e.g., pyrrhotite and pyrite) [64]. These sulfur reservoirs have been progressively oxidized due to mining operations or dissolved during long-term groundwater circulation/mixing, thereby directly supplying sulfate (SO_4_^2−^) to the system. In the DELOS area, deep fracture waters exhibit a highly dispersed hydrochemical character, which may primarily be the result of distinct water–rock interaction pathways controlled by different lithologies (e.g., gneiss-schistand granite), together with highly variable hydraulic connectivity and recharge sources. Distinct hydrochemical endmembers represent differentiated energy substrate pools, thereby selecting for and sustaining specific functional microbial assemblages.

The microbial community structures at all three locations demonstrate a pronounced environmental filtering effect [18,65,66] on dominant taxa. This demonstrates the close interaction between environmental conditions, functions and community compositions within deep fractured groundwater systems. In the KCO, groundwater is characterized by extreme salinity (TDS = 197,274 mg· L^−1^), long-term isolation from the surface (>100 million years), anoxia and severe oligotrophy [29]. These harsh conditions (Table S1) impose strong environmental filtering that selects for halophilic, obligate anaerobes capable of tolerating high osmotic pressure, resulting in a highly simplified community dominated by a single taxon, *Halanaerobiaeota* (family *Halobacteroidaceae*), at a relative abundance exceeding 92%. This family can adapt to hypersaline conditions by synthesizing compatible solutes (e.g., betaine) or regulating cellular membrane osmotic pressure, and can survive under anoxic conditions through fermentation and anaerobic chemoheterotrophic metabolism [67]. Most microorganisms are unable to tolerate such extremely high salinity and other harsh environmental conditions and are therefore excluded from competition, thereby creating a specialized ecological niche for *Halobacteroidaceae*. This pattern exemplifies community simplification, where extreme environments reduce niche breadth and eliminate all but the most specialized taxa [68,69]. In contrast, the SURF hosts moderately saline, sulphate-rich fracture fluids that span micro-oxic to anoxic conditions, favoring metabolically versatile *Gammaproteobacteria*, particularly the families *Hydrogenophilaceae*, *Rhodocyclaceae*, and *Halothiobacillaceae*, that are capable of sulfur oxidation, iron reduction, and facultative anaerobic respiration. *Pseudomonadota*, *Nitrospirota*, and *Verrucomicrobiota* co-dominate in DELOS, resulting in a multi-taxon co-dominant community structure. Multiple overlapping environmental gradients create a mosaic of microhabitats that promote niche partitioning among taxa. In shallow environments that are relatively oxic and characterized by low electrical conductivity, members of the phylum *Nitrospirota*, dominated by the chemolithoautotrophic genus *Leptospirillia*, can obtain energy through the oxidation of ferrous iron. In environments with high sulfate concentrations, communities are instead dominated by the class *Thermodesulfovibrionia* within *Nitrospirota*, which efficiently utilize sulfate as an electron acceptor. Certain lineages within Verrucomicrobiota are frequently associated with deep, oligotrophic environments [70], whereas Proteobacteria, owing to their high metabolic versatility, are widely distributed across different environmental gradients. This pattern underscores the role of spatial heterogeneity and niche differentiation [71] in regulating subsurface microbial biogeography under less extreme but more variable environmental conditions.

### 4.2. Significant Differences in the Microbial Community Composition Across the Three Aquifers Revealed by Bray–Curtis and Weighted Unifrac Distances

The hydrochemical characteristics of samples TM2848, TM4846, TM4752, and TM2794 (Figure 2) are consistent with their clustering patterns in the PCoA ordination (Figure 6a), further indicating that environmental filtering exerts a strong influence at these sampling sites. However, some samples (e.g., FractureA, FractureB, and FractureC) exhibit similar hydrochemical compositions but markedly different microbial community structures. This discrepancy may be attributed to the limited hydraulic communication among parallel fractures, whereby geographic isolation constrains microbial dispersal and results in community divergence over time or across permeable fractures through stochastic ecological processes [72,73]. The clustering of TM4348, TM2647, and TM901 from the DELOS region with the SURF sample in terms of weighted Unifrac distance reflects a high degree of evolutionary similarity in their microbial communities. At the phylum level, both groups share dominant microbial taxa such as *Pseudomonadota* and *Planctomycetota*, which results in their close proximity on the PCoA plot.

The differences between PCoA plots based on Bray–Curtis distance and weighted Unifrac distance stem from their distinct calculation logics. The Bray–Curtis distance focuses solely on the relative abundance of ASVs [74,75]. In contrast, the weighted Unifrac distance integrates both species abundance and phylogenetic relatedness, emphasizing the evolutionary divergence of communities [76]. This dual-perspective analysis underscores that deep subsurface microbial community structure is shaped by a combination of environmental filtering (driving abundance changes) and phylogenetic niche conservatism (driving evolutionary differentiation). Interestingly, although sample KCO appeared dissimilar to all others, the weighted Unifrac distance between KCO and DELOS was smaller than that between the two PI samples. This indicates that within-group differences can exceed between-group differences. Such “counter-intuitiveness” arises because weighted Unifrac incorporates not only relative abundances but also the phylogenetic relatedness of taxa, and it gives greater weight to highly abundant lineages. Moreover, the metric is highly sensitive to phylogenetic divergence at low taxonomic levels, which can produce a phenomenon that appears inconsistent with relative-abundance data.

### 4.3. Core Microbiome Is Present in Deep Groundwater Environments

The exploration of the core microbiome in the field of microbial ecology has become quite commonplace. However, discrepancies currently exist in how the core microbiome is defined and quantified [7]. Owing to methodological differences in data processing, cross-study comparisons often yield OTU-level Venn diagrams devoid of any overlap. Moreover, taxonomic assignments produced by one primer set may not map directly onto those generated by another, thereby imposing considerable challenges on the identification of a core microbiome across disparate datasets [77]. Therefore, finer classification levels are not necessarily preferable, a suitable classification level should be selected for analysis based on the actual circumstances of the samples. To study whether a core microbiome exists across the three selected aquifers, the defining criterion chosen was the common taxa across all sites [17,78,79].

According to the established defining criteria for the core microbiome, the core taxa of the three aquifers at different taxonomic levels are identified as follows: At the phylum level, six core phyla shared across all regions were identified, namely *Bacillota* (*Firmicutes*), *Actinomycetota* (*Actinobacteria*), *Pseudomonadota* (*Proteobacteria*), *Planctomycetota* (*Planctomycetes*), *Chloroflexota* (*Chloroflexi*), and *Deinococcota* (*Deinococcus-Thermus*). Among these, *Pseudomonadota* maintained the highest relative abundance in the microbial communities of all regions, exhibiting a significantly dominant position. At the genus level, seven core microbial genera were further screened out, including *Legionella*, *Lysobacter*, *Devosia*, *Shinella*, *Hyphomicrobium*, *Phreatobacter*, and *Pseudomonas*. As the dominant core phylum, *Pseudomonadota* and its subordinate core genera may play a key synergistic role in regulating the functions of the deep fracture water ecosystem (e.g., material cycling, environmental adaptability).

In some studies, the core microbiome is defined as the shared taxa across all samples (not all sites), which is a more stringent criteria because of the within-site heterogeneity. As shown in Table S4, only one family, *Comamonadaceae* (*Pseudomonadota*), was present in all 30 samples across the three regions. A few other families were present in >80% of the samples (i.e., high prevalence) and were among the top-10 families with the highest averaged relative abundance, including *Hydrogenophilaceae* (*Pseudomonadota*), *Omnitrophaceae* (*Verrucomicrobiota*), *BSV26* (*Candidatus Kryptonia*), and an unclassified *Thermodesulfovibrionia* (*Nitrospirota*).

### 4.4. The Functional Characteristics of Deep Fractures Are Collectively Shaped by Both Core Microbial Genera and Dominant Genera

Through the investigation of core microbial genera and their metabolic functions, we found that all seven core microbial genera are chemoheterotrophic bacteria, which is consistent with the dominance of chemoheterotrophy according to FAPROTAX analysis. Meanwhile, these genera primarily rely on organic carbon oxidation as their core metabolic process, with electron acceptors including O_2_ and NO_3_^−^. This corresponds to their occupation of distinct ecological niches: microaerophilic oligotrophy (*Phreatobacter*, *Legionella*), potential denitrification capacity (*Shinella*, *Hyphomicrobium*, *Devosia*), and metabolic versatility (*Pseudomonas*, *Lysobacter*). Due to the scarcity of KCO samples, their contribution to core microbial genera is fairly limited, and fermentation is presumably driven by the dominant genus *Fuchsiella*. This fermentative metabolism is consistent with the hypoxic and hypersaline hydrochemical characteristics of the deep aquifers in KCO. Iron respiration detected in Borehole GC is presumably derived from *Magnetospirillum*, a genus with relatively high abundance that is characterized by its capacity to sequester iron and produce the mineral magnetite (Fe_3_O_4_) under microaerobic environments [80]. This functional trait aligns with the detectable dissolved iron in Borehole GC groundwater [26]. The high abundance of human-pathogenic functions is likely associated with the pro-nounced presence of *Hydrogenophilaceae*, *Nitrosomonadaceae*, and *Rhodocyclaceae*. It is noteworthy that functional predictions at the family or genus level are insufficient to indicate actual pathogenic potential. Instead, metagenomic sequencing is required to verify the presence of genuine virulence genes. Meanwhile, it is necessary to consider environmental characteristics and the existence of potential human exposure pathways.

The high proportion of aerotrophic heterotrophs in certain areas reflects the microbial community’s adaptive strategy to this heterogeneity, wherein dominant microorganisms predominantly possess low-oxygen tolerance, enabling survival within the transition zone between aerobic and microaerophilic conditions underground. The relatively high abundance of this feature may also indicate the presence of material exchange or disturbance within the subsurface environment of the study area. Due to influences such as injection, production, or recharge, the deep subsurface system is not an isolated, closed system. On the other hand, nitrate respiration represents a prototypical anaerobic alternative respiration pathway. Its high abundance in synergy with other nitrogen metabolism functions indicates anaerobic conditions in the subsurface environment, further highlighting the spatial heterogeneity of subterranean ecosystems.

## 5. Conclusions

Through the meta-analysis of groundwater 16S rRNA gene amplicon sequencing data from the DELOS, the KCO and the SURF, we obtained an overall understanding of the deep-subsurface microbial diversity and functions across different countries/continents. Specifically, we found that the microbial community composition in deep, fractured aquifers is spatially heterogenous at the local (within site) and the global (between sites) scale, and even at the phylum level. A combined analysis of microbial community data and hydrochemical data suggests that environmental filtration is a key driver shaping the microbial community structure in deep groundwater environments. On the other hand, despite the spatial heterogeneity, a core microbiome exists among the three aquifers analyzed in this study, encompassing seven core microbial genera, namely *Legionella*, *Lysobacter*, *Devosia*, *Shinella*, *Hyphomicrobium*, *Phreatobacter*, and *Pseudomonas*. Only one family, *Comamonadaceae*, was present in all the 30 samples analyzed, whereas a few other prevalent families were found in >80% of the samples and had high averaged relative abundance, including *Hydrogenophilaceae* (*Pseudomonadota*), *Omnitrophaceae* (*Verrucomicrobiota*), *BSV26* (*Candidatus Kryptonia*), and an unclassified *Thermodesulfovibrionia* (*Nitrospirota*). Through functional prediction using FAPROTAX, it was found that chemoheterotrophic functions predominated. The coexistence of aerobic chemoheterotrophy and nitrate respiration demonstrates the spatial heterogeneity of oxygen content in deep subsurface environments, where the core microbial genera and dominant genera collectively govern the functional characteristics.

## Data Availability

The data presented in this study are available in [European Nucleotide Archive at the European Bioinformatics Institute] at [https://www.ebi.ac.uk/ena/browser/home, accessed 15 September 2025], reference number [PRJEB35125; PRJEB44691; PRJEB49237]; [National Center for Biotechnology Information’s (NCBI)] at [https://www.ncbi.nlm.nih.gov/, accessed 15 September 2025], reference number [PRJNA1181539]. These data were derived from the following resources available in the public domain: [list European Nucleotide Archive at the European Bioinformatics Institute, https://www.ebi.ac.uk/ena/browser/home and National Center for Biotechnology Information’s (NCBI), https://www.ncbi.nlm.nih.gov/].

## Supplementary Materials

The following supporting information can be downloaded at: https://www.mdpi.com/article/10.3390/microorganisms14010045/s1, Figure S1: Full legend for the relative abundance plots in Figure 3 of the main manuscript. Figure S2: The microbial community composition of 30 samples at the phylum level. Table S1: Regional hydrochemical data for SURF, DELOS and KCO. Table S2: The number of raw reads, the library size after quality filtering, and the number of ASVs. Table S3: Names of the phyla in each compartment of the phylum-level Venn diagram (Figure 7). Table S4: ASV table agglomerated to the Family level for the 30 samples analyzed.
