# Supplementary material for: Multi-Regional Study on the Microbial Community Structure, Core Microbiome and Functional Characteristics in Deep Fracture Waters
Source: Microorganisms. 2025 Dec 25;14(1):45. doi: 10.3390/microorganisms14010045 (PMC12844516; doi:10.3390/microorganisms14010045)
Supplement: Supplementary file 1 [file microorganisms-14-00045-s001.zip › Microorganisms_SI.pdf]

microorganisms

Supporting Information for

Multi-Regional Study on the Microbial Community Structure, Core Microbiome and Functional Characteristics in Deep Fracture Waters

Xiaoxuan Li [1,2], Tianming Huang [1,2], Yiman Li [1,2], Zhonghe Pang [1,2] and Yuran Zhang [1,2,*]

[1] State Key Laboratory of Lithospheric and Environmental Coevolution, Institute of Geology and Geophysics, Chinese Academy of Sciences, Beijing 100029, China
[2] College of Earth and Planetary Sciences, University of Chinese Academy of Sciences, Beijing 101408, China
* Correspondence: yuranzhang@mail.iggcas.ac.cn

Contents:

This SI includes Figure S1, Figure S2 and a Microsoft Excel file called the Supplementary Tables S1-S4.

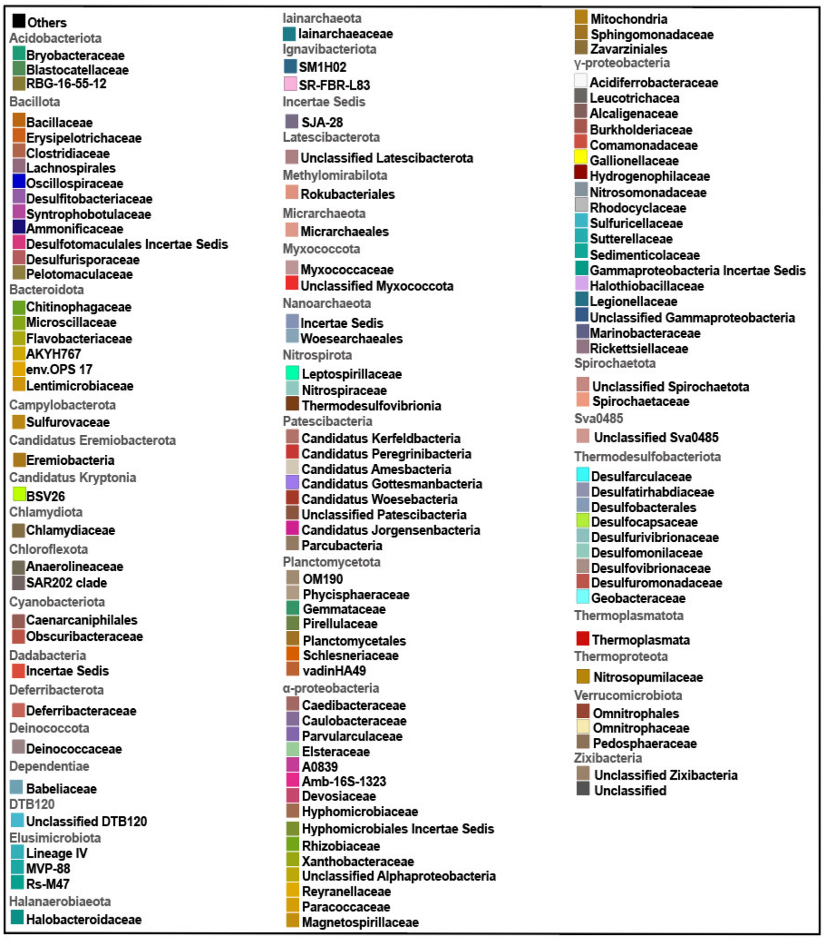

Figure S1. Full legend for the relative abundance plots in Figure 3 of the main manuscript.

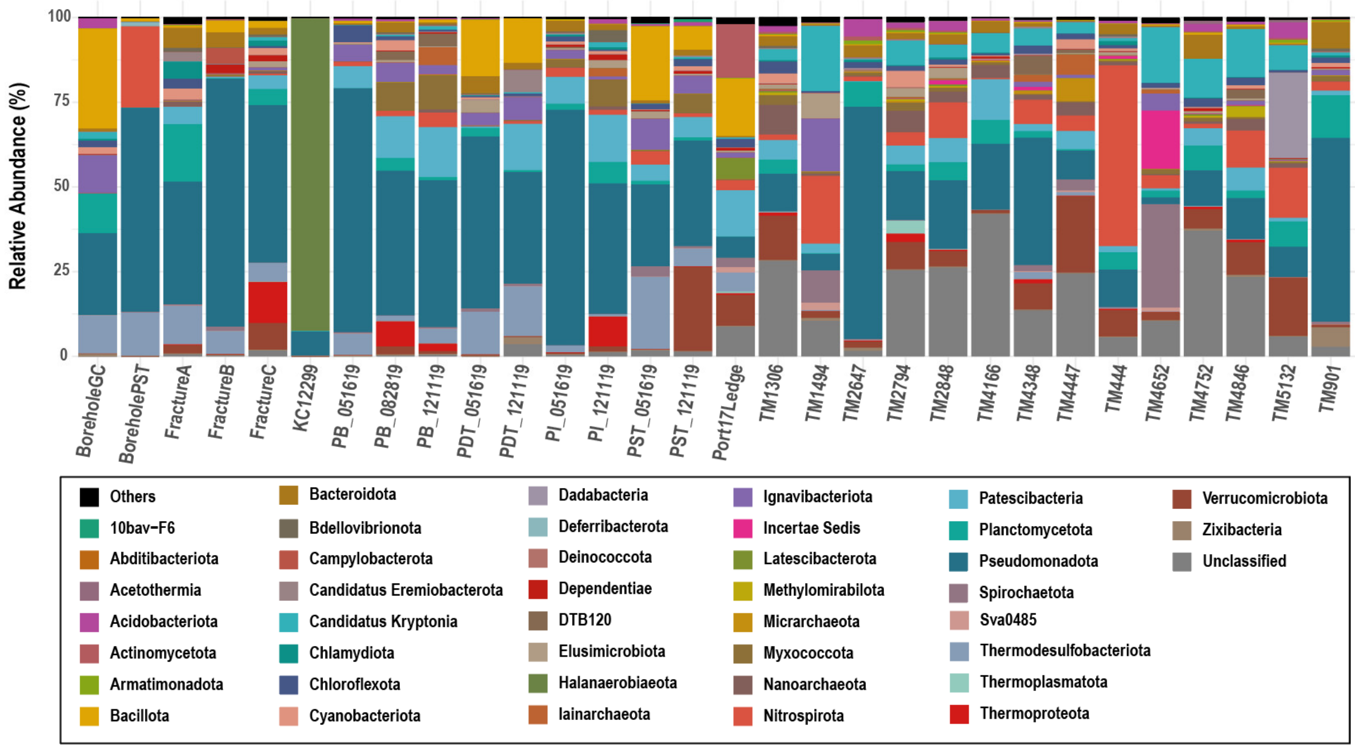

Figure S2. The microbial community composition of 30 samples. Bar plots show the finest classification possible at the phylum level. The major taxa (i.e., taxa that were within the top 10 most abundant in at least one sample) are shown in color.

Supplementary Tables S1-S4

Table S1. Regional hydrochemical data.

Table S2. The number of raw reads, the library size after quality filtering, and the number of ASVs.

Table S3. Names of the phyla in each compartment of the phylum-level Venn diagram (Figure 7).

Table S4. ASV table agglomerated to the Family level for the 30 samples analyzed.
